# Association of alcohol consumption with all‐cause mortality, new‐onset stroke, and coronary heart disease in patients with abnormal glucose metabolism—Findings from a 10‐year follow‐up of the REACTION study

**DOI:** 10.1111/1753-0407.13371

**Published:** 2023-04-11

**Authors:** Mengzhao Cui, Fei Li, Xiaokun Gang, Yuan Gao, Xianchao Xiao, Gang Wang, Yujia Liu, Guixia Wang

**Affiliations:** ^1^ Department of Endocrinology and Metabolism The First Hospital of Jilin University Jilin China

**Keywords:** alcohol consumption, health outcomes, lifestyles, longitudinal study, 饮酒, 健康结果, 生活方式, 纵向研究

## Abstract

**Background:**

Type 2 diabetes mellitus (T2DM) and diabetic complications threaten human health seriously. Healthy lifestyles can lower the risk of cardiovascular disease (CVD) and long‐term complications. However, the relationship between alcohol consumption and CVD mortality is still controversial, and there is a lack of evidence from large‐scale longitudinal studies in the Chinese population. Based on the REACTION study (Risk Evaluation of Cancers in Chinese Diabetic Individuals: A Longitudinal Study), this paper explores the association between alcohol consumption and all‐cause mortality, stroke, and coronary heart disease (CHD) in patients with abnormal glucose metabolism during a 10‐year follow‐up period to provide evidence for lifestyle counselling for these patients.

**Methods:**

First, baseline data were collected from the REACTION study cohort in Changchun, Jilin Province, China, in 2011–2012. A questionnaire survey was performed among patients with abnormal glucose metabolism aged over 40 years. The frequency of their alcohol intake, the type of alcohol, and the amount of alcohol consumed daily were surveyed. Physical and biochemical examinations were also performed. Then, through the Primary Public Health Service System of Jilin Province, we collected outcomes during the 10‐year follow‐up up to October 1, 2021, including all‐cause mortality, stroke, and CHD. Next, we conducted logistic regression to analyze the relationship between baseline alcohol consumption and 10‐year outcomes, and risk ratio (RR) and 95% CI were calculated by adjusting for different clinical indicators. A *p* value < 0.05 was considered statistically significant.

**Results:**

A total of 4855 patients with T2DM and prediabetes (35.2% men and 64.8% women) were included in the baseline analysis. Outcomes of 3521 patients during the 10‐year follow‐up were obtained, including 227 deaths, 296 new‐onset strokes and 445 new‐onset CHD. Occasional drinking (less than once a week) was associated with a reduced 10‐year all‐cause mortality, with an RR of 0.511 (95% CI [0.266, 0.982]) after adjustment for age, gender, medical history, and lifestyles and an RR of 0.50 (95% CI [0.252, 0.993]) in a fully adjusted model including additional biochemical indicators. In addition, heavy alcohol consumption (≥30 g/day for men and ≥15 g/day for women) was significantly associated with an increased incidence of stroke, with an RR of 2.503 (95% CI [1.138, 5.506]) after the adjustment for age, gender, medical history, lifestyles, and biochemical indicators. No significant association was found between alcohol consumption and new‐onset CHD.

**Conclusions:**

For patients with abnormal glucose metabolism, occasional drinking (less than once a week) reduces the risk of all‐cause mortality, while heavy alcohol consumption (≥30 g/day for men and ≥15 g/day for women) significantly increases the risk of new‐onset stroke. They should avoid heavy alcohol intake, but light alcohol consumption or occasional drinking is acceptable. Additionally, it is crucial to control blood glucose and blood pressure and keep performing physical activities.

## Graphical abstract

**Highlights**

- This long‐term longitudinal study demonstrates the association between alcohol consumption and health outcomes in patients with abnormal glucose metabolism including type 2 diabetes mellitus and prediabetes.
- Occasional drinking (less than once a week) reduces the risk of all‐cause mortality, while heavy alcohol consumption (≥30 g/day for men and ≥15 g/day for women) significantly increases the risk of new‐onset stroke.
- Patients with abnormal glucose metabolism should avoid heavy alcohol intake, but light alcohol consumption or occasional drinking is acceptable.
- This study provided evidence of lifestyle instructions for patients with abnormal glucose metabolism.

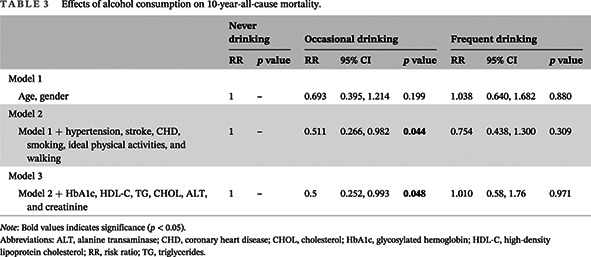

## 摘要

**目的:**2型糖尿病(T2DM)及其并发症严重威胁人类健康。健康的生活方式可以降低心血管疾病(CVD)和长期并发症的风险。然而, 饮酒与心血管疾病死亡率之间的关系仍存在争议, 缺乏对中国人群进行大规模纵向研究的证据。本文以中国糖尿病患者癌症风险评估纵向研究(REACTION)为基础, 对糖代谢异常患者进行10年随访, 探讨饮酒与全因死亡率、卒中及冠心病的关系, 为其提供生活方式指导依据。

**方法:**首先, 基线数据收集自2011‐2012年中国吉林省长春市的REACTION研究队列, 对40岁以上糖代谢异常患者进行问卷调查, 调查了他们的饮酒频率、饮酒类型和每日饮酒量, 并对其进行了生理生化检查。然后, 通过吉林省初级公共卫生服务系统, 我们收集了截至2021年10月1日的10年随访结果, 包括全因死亡率、中风和冠心病。随后, 我们进行logistic回归分析基线饮酒与10年预后之间的关系, 并通过调整不同临床指标计算风险比(RR)和95%CI。P<0.05为有统计学意义。

**结果:**共有4855例2型糖尿病和前驱糖尿病患者(男性35.2%, 女性64.8%)被纳入基线分析。在10年的随访中获得了3521例患者的结果, 包括227例死亡, 296例新发卒中和445例新发冠心病。偶尔饮酒(每周少于1次)与10年全因死亡率降低相关, 经年龄、性别、病史和生活方式调整后, RR为0.511(95%CI: 0.266,0.982), 在包括额外生化指标的完全调整模型中, RR为0.50 (95%CI: 0.252,0.993)。此外, 大量饮酒(男性≥30 g/天, 女性≥15 g/天)与卒中发病率增加显著相关, RR为2.503 (95%CI:1.138, 5.506), 调整年龄、性别、病史、生活方式和生化指标。饮酒与新发冠心病之间未发现显著相关性。

**结论**:对于糖代谢异常患者, 偶尔饮酒(每周< 1次)可降低全因死亡风险, 而大量饮酒(男性≥30 g/d, 女性≥15 g/d)可显著增加新发脑卒中风险。应该避免大量饮酒, 但少量饮酒或偶尔饮酒是可以接受的。此外, 控制血糖和血压, 坚持体育锻炼也很重要。

## 1 INTRODUCTION

The past 30 years have seen a growing trend of diabetes mellitus (DM) and prediabetes in China. The prevalence of DM in Chinese adults was 4.5% in urban areas and 1.8% in rural areas in 2002, while it rose to 11.2% in 2015–2017. Furthermore, in 2010, 493.4 million adults (50.1% of the total population in China) had prediabetes.^1^ DM and diabetic complications pose a great threat to human health. So far, population‐based longitudinal studies have consistently suggested that type 2 diabetes mellitus (T2DM) is associated with risk of total cardiovascular disease (CVD), mainly coronary heart disease (CHD) and stroke.^2^, ^3^ Thus, prevention and treatment of DM are particularly important. It is well‐known that healthy lifestyles are significant for patients with DM and prediabetes. The relationship between alcohol consumption and CVD mortality has raised concern worldwide. In the Global Burden of Disease (GBD) Study from 2019, alcohol use is the ninth leading risk factor for morbidity and mortality.^4^ Several studies have reported that light‐to‐moderate alcohol consumption in the general population was associated with a decrease in CHD^5^, ^6^ and all‐cause mortality.^7^, ^8^, ^9^ In contrast, some studies indicated that low to moderate alcohol consumption had no cardioprotective effect on Chinese and Indians,^10^, ^11^ and regular alcohol intake increased all‐cause mortality.^12^ For patients with DM, the effect of alcohol consumption on health outcomes remains controversial. In the ADVANCE study, moderate alcohol use, particularly wine consumption, was associated with reduced risks of cardiovascular events and all‐cause mortality in patients with T2DM.^13^ The ACCORD trial suggested that moderate alcohol consumption was associated with hypertension and might elevate cardiovascular risks.^14^ Recently, a cross‐sectional study in China indicated that drinking 1–3 days per week or daily was negatively related to stroke.^15^ There is still a lack of long‐term longitudinal studies in China about the impact of alcohol consumption on health outcomes, including all‐cause mortality, stroke, and CHD in patients with abnormal glucose metabolism including DM and prediabetes. The frequency and quantity of alcohol consumption need to be considered in such studies. This paper aims to investigate the association between alcohol consumption and all‐cause mortality, stroke, and CHD in patients with abnormal glucose metabolism to provide them with guidance for alcohol consumption.

## 2 METHODS

### 2.1 Subjects

The baseline survey was a part of the Risk Evaluation of Cancers in Chinese Diabetic Individuals: A Longitudinal Study (REACTION)^16^ in China. Subjects aged over 40 years were recruited in Changchun City from 2011–2012. In the present study, we selected subjects who met the diagnostic criteria of T2DM and prediabetes proposed by the World Health Organization (WHO) in 1999.^17^ The outcomes during the 10‐year follow‐up period ending October 1, 2021, including death, stroke, and CHD, were collected from the Primary Public Health Service System of Jilin Province. Our study complies with the Declaration of Helsinki and was approved by the ethics committee of the First Hospital of Jilin University. All subjects provided written informed consent before this study.

### 2.2 Exclusion criteria

Patients with (1) acute stress reactions such as infection, trauma, and surgery; (2) pregnancy; and (3) a recent history of fever were excluded from this study. Furthermore, we excluded stroke at baseline when the target outcome was stroke and excluded CHD at baseline when the target outcome was CHD.

### 2.3 Questionnaire survey

Basic information, including name, gender, age, medical history, smoking and drinking history, physical activity (including high‐intensity activity, moderate‐intensity activity, and walking), and sedentary behaviors, was obtained by trained physicians or public health workers.

### 2.4 Physical examination

Height and body weight were measured according to a standard protocol, and body mass index (BMI) was calculated as body weight in kilograms divided by the square of height in meters.

### 2.5 Laboratory examination

After an overnight fast of at least 8 h, the levels of fasting plasma glucose (FPG), 2‐h postprandial plasma glucose (PPG), serum lipid, and glycosylated hemoglobin (HbA1c) were measured, and the hepatic function and renal function were evaluated.

### 2.6 Evaluation criteria

#### 2.6.1 Smoking

Subjects who smoked at least one cigarette per day or seven cigarettes per week for more than 6 months were defined as “smokers.” Individuals who never smoked or smoked occasionally were defined as “nonsmokers.”

#### 2.6.2 Alcohol consumption

Alcohol intake was classified into three categories based on frequency: (1) no alcohol consumption, (2) occasional alcohol consumption (less than once a week), and (3) frequent alcohol consumption (at least once a week). Based on the amount of drinking, alcohol consumption was classified into (1) light alcohol consumption (<5 g/day), (2) moderate alcohol consumption (5–29.9 g/day for men and 5–14.9 g/day for women), and (3) heavy alcohol consumption (≥30 g/day for men and 15 g/day for women) (amount [g/d] = daily alcohol volume [mL] × alcoholicity [V/V] × density [g/mL]; density of alcohol was calculated as 0.8 g/mL).^18^ Three types of alcohol were included, namely, liqueur, beer, and red wine.

### 2.7 Physical activity

Ideal physical activities were defined as ≥150 min/week at moderate intensity, ≥75 min/week at vigorous intensity, or ≥150 min/week at moderate and vigorous intensity.^19^ All forms of walking were considered, including walking during work and spare time that lasted at least 10 min.

### 2.8 Sedentariness

Sedentariness refers to the state of being sedentary for an average time of ≥30 h per week.

### 2.9 Determination of outcomes

We obtained annual physical examination results recorded by physicians in the community in the Primary Public Health Service System of Jilin Province. We assessed the outcomes, including all‐cause mortality, stroke (including ischemic and hemorrhagic stroke), and CHD, and outcomes that occurred after baseline were identified according to the first record in the system.

### 2.10 Statistical analysis

Baseline data were grouped by gender. Continuous variables which were abnormally distributed were represented as medians (25th and 75th percentiles), and categorical variables were shown by counts and percentages. Differences between the two groups were analyzed by the Mann–Whitney *U* test and Pearson chi‐square test, respectively. Logistic regression was conducted to analyze the relationship between alcohol consumption at baseline and the risk of 10‐year all‐cause mortality, new‐onset stroke, and CHD. Baseline age, gender, disease history, lifestyles, and biochemical examination results were further adjusted for models. Other baseline risk factors associated with 10‐year outcomes were also analyzed. Risk ratio (RR) and 95% CI were calculated for each outcome. As for the missing values, we used the *regression imputation* method to replace the missing values for the categorical variables and used the *dummy variable* method to replace the missing continuous variables. All reported *p* values were two‐sided, and *p* < 0.05 was considered statistically significant. SPSS 24.0 was used for statistical analysis, and GraphPad Prism 7.0 was used for forest mapping.

## 3 RESULTS

### 3.1 Baseline characteristics of subjects

The baseline characteristics of subjects are summarized in Table 1. A total of 4855 subjects were included in the baseline analysis (35.2% men and 64.8% women). Of these, 4.3% data on drinking, 5.4% data on smoking, 1.0% data on history of CHD, and less than 1% of other data were missing. History of stroke, smoking, drinking, physical activity, sedentariness, BMI, FPG, creatinine, alanine transaminase (ALT), aspartate aminotransferase (AST), and gamma‐glutamyl transpeptidase (GGT) were significantly higher in men than in women. In contrast, history of CHD, cancer, high‐density lipoprotein cholesterol (HDL‐C), low‐density lipoprotein cholesterol (LDL‐C), cholesterol, and triglycerides (TG) were significantly lower in men than in women.

**TABLE 1:** Baseline characteristics of subjects.

Clinical information	Men (*n* = 1708)	Women (*n* = 3147)	*p* value
Hypertension	352 (25.6%)	702 (27.2%)	0.277
Stroke	72 (4.3%)	79 (2.5%)	0.001
CHD	195 (11.5%)	496 (16.0%)	<0.001
Cancer	26 (1.6%)	157 (5.2%)	<0.001
Smoking	446 (28.5%)	288 (10.0%)	<0.001
Drinking			<0.001
Light alcohol consumption	1080 (71.4%)	2843 (99.0%)	
Moderate alcohol consumption	91 (6.0%)	10 (0.3%)	
Heavy alcohol consumption	342 (22.6%)	20 (0.7%)	
Ideal physical activity	193 (13.2%)	218 (8.2%)	<0.001
High intensity	100 (6.9%)	118 (4.5%)	0.001
Moderate intensity	172 (11.9%)	213 (8.1%)	<0.001
Walking	1071 (72.6%)	2010 (72.1%)	0.734
Sedentariness	559 (37.7%)	959 (34.5%)	0.035
Age (years)	67.4 (60.0, 73.3)	66.0 (59.8, 71.6)	0.669
BMI (kg/m^2^)	25.94 (23.83, 28.04)	25.39 (23.42, 27.68)	<0.001
HbA1c (%)	6.2 (5.8, 7.1)	6.2 (5.9, 7.0)	0.069
FPG (mmol/L)	6.1 (5.48, 7.27)	5.95 (5.41, 6.9)	<0.001
PPG (mmol/L)	8.93 (6.86, 12.68)	8.88 (7.09, 12.11)	0.639
Creatinine (μmol/L)	75.6 (69.2, 84.5)	62.8 (58.4, 68.2)	<0.001
HDL‐C (mmol/L)	1.14 (0.99, 1.33)	1.24 (1.09, 1.43)	<0.001
LDL‐C (mmol/L)	2.85 (2.35, 3.35)	3.01 (2.54, 3.53)	<0.001
CHOL (mmol/L)	4.96 (4.35, 5.61)	5.27 (4.7, 5.91)	<0.001
TG (mmol/L)	1.58 (1.10, 2.42)	1.70 (1.21, 2.42)	0.005
ALT (U/L)	14.0 (11.0, 20.0)	12.0 (9.0, 17.0)	<0.001
AST (U/L)	20.0 (17.0, 25.0)	19.0 (17.0, 23.0)	<0.001
GGT (U/L)	30.0 (21.0, 47.0)	22.0 (16.0, 32.0)	<0.001

Abbreviations: ALT, alanine transaminase; AST, aspartate aminotransferase; BMI, body mass index; CHD, coronary heart disease; CHOL, cholesterol; FPG, fasting plasma glucose; GGT, gamma‐glutamyl transpeptidase; HbA1c, glycosylated hemoglobin; HDL‐C, high‐density lipoprotein cholesterol; LDL‐C, low‐density lipoprotein cholesterol; PPG, postprandial plasma glucose; TG, triglycerides.

### 3.2 Distribution of 10‐year follow‐up outcomes grouped by gender and age

The 10‐year follow‐up outcomes of the 3521 subjects were obtained from the Primary Public Health Service System of Jilin Province. As shown in Table 2, the outcomes included 227 deaths, 296 new‐onset strokes, and 445 new‐onset CHD. For both men and women, the incidence of the above outcomes, except CHD, increased with age.

**TABLE 2:** Distribution of 10‐year‐follow‐up outcomes grouped by gender and age.

	Death		Stroke		CHD	
	Men	Women	Men	Women	Men	Women
Age (years)						
<50	2 (1.23%)	4 (1.44%)	3 (1.84%)	9 (3.25%)	14 (8.59%)	24 (8.66%)
50–59	18 (4.42%)	15 (1.84%)	25 (6.14%)	57 (6.99%)	58 (14.25%)	115 (14.09%)
60–69	47 (11.41%)	56 (6.24%)	42 (10.19%)	84 (9.35%)	57 (13.83%)	124 (13.81%)
70–79	31 (16.49%)	43 (13.27%)	26 (13.83%)	44 (13.58%)	21 (11.17%)	28 (8.64%)
≥80	6 (31.58%)	5 (29.41%)	3 (15.79%)	3 (17.65%)	1 (5.26%)	2 (11.76%)

Abbreviation: CHD, coronary heart disease.

### 3.3 Effects of alcohol consumption on 10‐year follow‐up outcomes

#### 3.3.1 All‐cause mortality

Compared with never drinking, occasional drinking was more significantly associated with a reduced 10‐year all‐cause mortality, with an RR of 0.511 (95% CI [0.266, 0.982]) after adjustment for age, gender, disease history, smoking, ideal physical activities, and walking. A significant association was observed in fully adjusted models considering age, gender, disease history, smoking, ideal physical activities, walking, and serum biochemical indicators, with an RR of 0.50 (95% CI [0.252, 0.993]) (Table 3).

**TABLE 3:** Effects of alcohol consumption on 10‐year‐all‐cause mortality.

	Never drinking		Occasional drinking			Frequent drinking		
	RR	*p* value	RR	95% CI	*p* value	RR	95% CI	*p* value
Model 1								
Age, gender	1	–	0.693	0.395, 1.214	0.199	1.038	0.640, 1.682	0.880
Model 2								
Model 1 + hypertension, stroke, CHD, smoking, ideal physical activities, and walking	1	–	0.511	0.266, 0.982	**0.044**	0.754	0.438, 1.300	0.309
Model 3								
Model 2 + HbA1c, HDL‐C, TG, CHOL, ALT, and creatinine	1	–	0.5	0.252, 0.993	**0.048**	1.010	0.58, 1.76	0.971

*Note*: Bold values indicates significance (*p* < 0.05).

Abbreviations: ALT, alanine transaminase; CHD, coronary heart disease; CHOL, cholesterol; HbA1c, glycosylated hemoglobin; HDL‐C, high‐density lipoprotein cholesterol; RR, risk ratio; TG, triglycerides.

#### 3.3.2 Stroke

Compared with light alcohol consumption, heavy alcohol consumption was more significantly associated with an increased risk of new‐onset stroke. The 10‐year RR of model 1, model 2, and model 3 was 2.326 (95% CI [1.088, 4.975]), 2.474 (95% CI [1.124, 5.445]), and 2.503 (95% CI [1.138, 5.506]), respectively (Table 4).

**TABLE 4:** Effects of alcohol consumption on 10‐year new‐onset stroke.

	Light alcohol consumption		Heavy alcohol consumption		
	RR	*p* value	RR	95% CI	*p* value
Model 1					
Age, gender	1	–	2.326	1.088, 4.975	**0.03**
Model 2					
Model 1 + hypertension, CHD, smoking, ideal physical activities, walking, and sedentariness	1	–	2.474	1.124, 5.445	**0.024**
Model 3					
Model 2 + HbA1c, HDL‐C, TG, CHOL, ALT, and creatinine	1	–	2.503	1.138, 5.506	**0.023**

*Note*: Bold values indicates significance (*p* < 0.05).

Abbreviations: ALT, alanine transaminase; CHD, coronary heart disease; CHOL, cholesterol; HbA1c, glycosylated hemoglobin; HDL‐C, high‐density lipoprotein cholesterol; RR, risk ratio; TG, triglycerides.

Furthermore, we found no significant association between alcohol consumption and new‐onset CHD (*p* > 0.05).

### 3.4 Association of baseline risk factors with 10‐year outcomes

**FIGURE 1:**
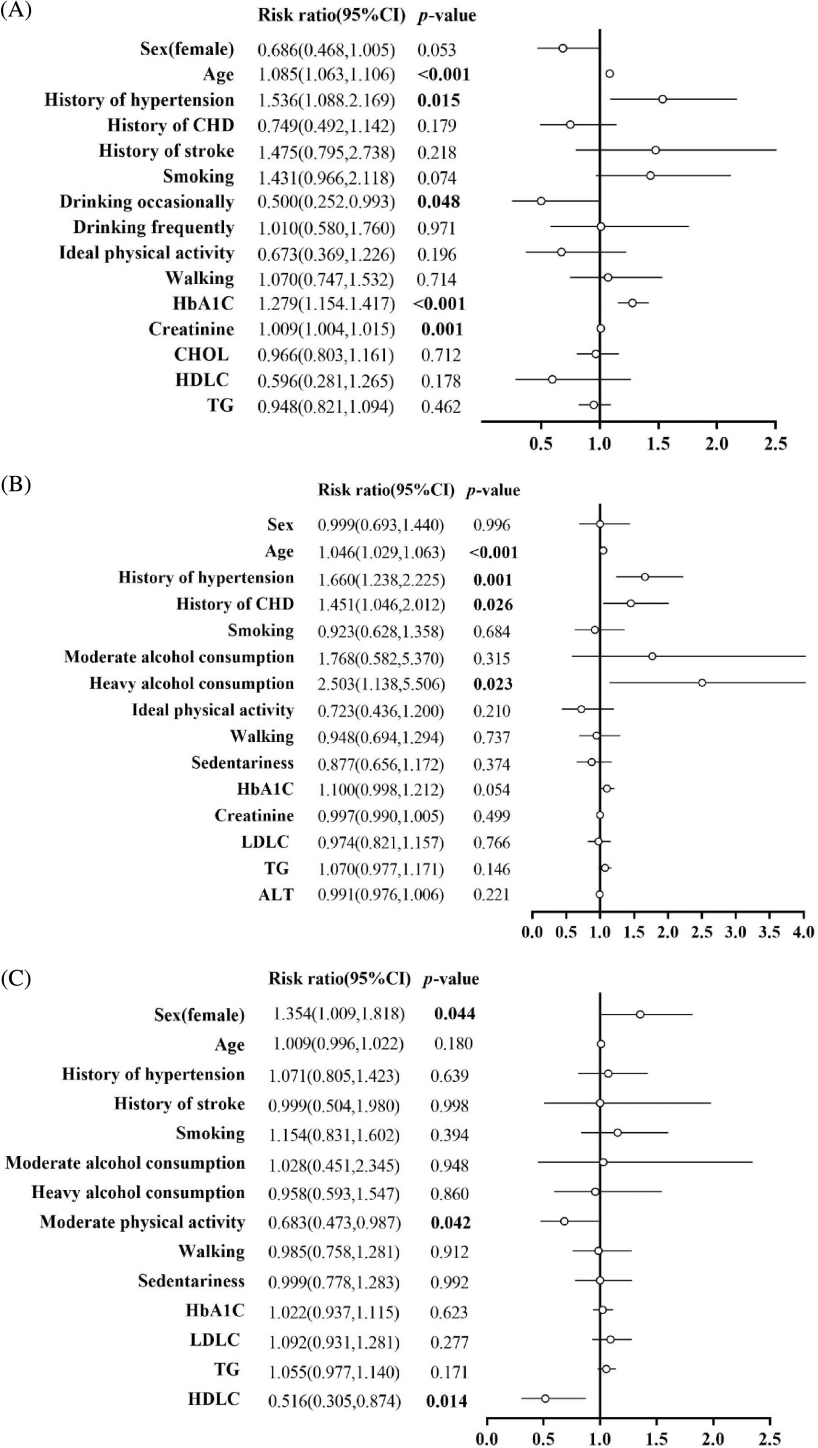
(A) Association of baseline risk factors with all‐cause mortality. (B) Association of baseline risk factors with new‐onset stroke. (C) Association of baseline risk factors with new‐onset CHD.

Increased age (RR 1.085, 95% CI [1.063, 1.106]), history of hypertension (1.536 [1.088, 2.169]), elevated HbA1c (1.279 [1.154, 1.417]), and creatinine (1.009 [1.004, 1.015]) were associated with all‐cause mortality (Figure 1A). Increased age (1.046 [1.029, 1.063]), history of hypertension (1.660 [1.238, 2.225]), and history of CHD (1.451 [1.046, 2.012]) were associated with new‐onset stroke (Figure 1B). Female gender (1.354 [1.009, 1.818]) was associated with new‐onset CHD, while moderate‐intensity activities (0.683 [0.473, 0.987]) and higher HDL‐C (0.516 [0.305, 0.874]) were associated with a lower risk of CHD (Figure 1C).

## 4 DISCUSSION

To our knowledge, this is the first long‐term observational study focusing on the association between alcohol consumption and CVD and all‐cause mortality in China. We conducted a 10‐year longitudinal study on Chinese people with abnormal glucose metabolism and suggested the impact of alcohol consumption on health outcomes. We found that compared with never drinking, occasional drinking was more significantly associated with a reduced risk of all‐cause mortality, with an RR of 0.50 (95% CI [0.252, 0.993]) in fully adjusted models. Furthermore, compared with light consumption, heavy alcohol consumption was more significantly associated with increased new‐onset stroke (RR 2.503, 95% CI [1.138, 5.506]). No significant association was found between alcohol consumption and new‐onset CHD. Age, history of hypertension, HbA1c, and creatinine levels were positively associated with all‐cause mortality. Age, history of hypertension and CHD were positively associated with new‐onset stroke. Female gender was associated with increased new‐onset CHD, while moderate‐intensity activities and higher HDL‐C were associated with a lower risk of CHD.

On the one hand, we chose the population with glucose metabolism disorders because the guidelines for diabetes provided few instructions on alcohol consumption. Most previous studies have shown that drinking was harmful for individuals with diabetes, and we often instructed them to limit alcohol consumption. On the other hand, the impact of alcohol consumption on health outcomes, especially mortality, stroke, and CHD, was still controversial. In the present longitudinal study, we sought to determine whether alcohol consumption was harmful and how much alcohol consumption was appropriate for people with abnormal glucose metabolism.

The ADVANCE study^13^ was conducted in 20 European countries. However, alcohol consumption in Asia was significantly lower than in European countries. Therefore, the results of the ADVANCE study may only be specific to Europeans and had no reference value for studies on Chinese people. In addition, the analyses were based on alcohol consumption at the time of randomization in the ADVANCE trial, so the actual alcohol consumption might be disturbed by the grouping. In a Danish cross‐sectional study,^20^ among people with diabetes, high alcohol consumption (>21 drinks/week for men and >14 drinks/week for women) was more significantly associated with a lower odds ratio of myocardial infarction and stroke compared to low alcohol consumption, but this finding is only specific to men. Another cross‐sectional study suggested that low‐to‐moderate alcohol intake seemed to be associated with reduced prevalence of acute coronary syndrome (ACS), whereas high alcohol consumption was associated with increased levels of lipids and blood pressure and increased risk of ACS.^21^ Our study analyzed the impact of alcohol consumption on all‐cause mortality, stroke, and CHD based on a longitudinal observation. The results are more reliable than those in cross‐sectional studies. The different results of various studies can be attributed to genetic variations and differences in ethnic origin, and the definition and grouping of alcohol consumption.

The mechanisms of the adverse effects of heavy alcohol consumption on stroke are as follows: Excessive alcohol intake may elevate the level of blood pressure and TG in people with diabetes,^22^ resulting in cerebrovascular diseases.^23^ Furthermore, heavy alcohol consumption adversely affects brain structure and function, causing atrophy of the hippocampus and cerebellum.^24^ A previous study found that there was a dose–response relationship between alcohol consumption and lower extremity arterial disease in patients with diabetes.^25^ It was also reported that alcohol intake was positively correlated with peripheral atherosclerotic plaque volume,^26^ suggesting that heavy consumption may result in macrovascular diseases. Recently, it was reported that alcohol‐induced imbalances in the symbiotic relationship between the host and the gut microbiota could lead to the development and progression of CVD.^27^ In the present study, we also found that occasional drinking could more significantly decrease all‐cause mortality compared with never drinking, indicating that drinking less than once a week may have a positive effect on health outcomes. However, no association was found between light or moderate alcohol consumption and the outcomes. One of the reasons is that the major cases of new‐onset CHD in our study were female, and most of them drank less alcohol than males. It was reported that light‐to‐moderate alcohol intake may augment insulin sensitivity, raise HDL‐C, suppress inflammation, raise adiponectin, and improve endothelial function.^28^, ^29^ Additionally, angiogenic effects of moderate alcohol consumption were concomitant with other beneficial cardio‐protective effects, including improved microvascular reactivity, endothelial function, and myocardial perfusion in the ischemic regions of the myocardium.^30^, ^31^

The present study has several limitations. First, the follow‐up outcomes were obtained from the service system, and the data might not be accurate. The information on alcohol consumption was self‐reported, and recall bias could exist. Second, we did not take abstainers into consideration. Instead, we calculated the amount of alcohol consumed as long as the subject drank. Third, subjects with T2DM and prediabetes were not analyzed separately, so we could not know whether the association between alcohol consumption and health outcomes was the same in these two cases. In the future, we will expand the population size, collect more information and clinical data, and refine the grouping. Forth, because we acquired the outcomes 10 years after baseline, we could not know the changes in alcohol consumption over time and the baseline variables that could change with time. We will focus on the effect of time on the association and get relatively complete clinical data in future work. In addition, in‐depth studies will be conducted to investigate the essential association between alcohol consumption and CHD, stroke, and other outcomes.

## 5 CONCLUSION

This study demonstrates the association between alcohol consumption and health outcomes of patients with abnormal glucose metabolism in China. Occasional drinking (less than once a week) decreases all‐cause mortality, while heavy alcohol consumption (≥30 g/day for men and ≥15 g/day for women) increases the prevalence of stroke. Heavy alcohol consumption causes CVD, while occasional or light alcohol consumption improves the detrimental outcomes. Patients with abnormal glucose metabolism should avoid heavy alcohol intake, but they should not be prevented from occasional or light alcohol consumption. In addition, it is crucial for them to control blood glucose and blood pressure and keep performing physical activities.

## AUTHOR CONTRIBUTIONS

Y.L., G.W., and X.G. contributed to the conception and design of the study. Y.G., X.X., G.W., F.L. and Y.L. conducted the baseline investigation of this study. M.C. and F.L. collected the events of the 10‐year follow‐up. Y.G. organized the database. X.X. performed the statistical analysis. M.C. wrote the first draft of the manuscript. X.G. revised the manuscript. F.L. gave diet and exercise introductions to patients with diabetes and prediabetes. All authors contributed to manuscript revision and read and approved the submitted version.

## FUNDING INFORMATION

This study was funded by the Department of Science and Technology of Jilin Province (China) (20210303001SF and 20170623005TC) and Finance Department of Jilin Province (China) (JLSWSRCZX2021‐075).

## CONFLICT OF INTEREST STATEMENT

Mengzhao Cui, Fei Li, Xiaokun Gang, Yuan Gao, Xianchao Xiao, Gang Wang, Yujia Liu, and Guixia Wang declare that they have no conflict of interest.
